# Job Crafting: A Challenge to Promote Decent Work for Vulnerable Workers

**DOI:** 10.3389/fpsyg.2021.681022

**Published:** 2021-05-20

**Authors:** Andrea Svicher, Annamaria Di Fabio

**Affiliations:** Department of Education, Languages, Intercultures, Literatures and Psychology (Psychology Section), University of Florence, Florence, Italy

**Keywords:** job crafting, decent work, vulnerable workers, psychology of working theory, human rights

## Abstract

In recent years, the decent work agenda has called upon vocational psychologists to advance psychological research and intervention to promote work as a human right. Furthermore, the COVID-19 pandemic is having disproportionate consequences on vulnerable workers, such as unemployment and underemployment, highlighting the need to enhance access to decent work for these workers. As a response, the present perspective article advances job crafting as a promising way to shape decent work for marginalized workers. To this end, the article deals with decent work and job crafting, starting with the definition of decent work according to the psychology of working theory (PWT) and examining the evolution of the construct of job crafting. Subsequently, the literature on job crafting is discussed, focusing on variables related to the PWT model of decent work and their effect on vulnerable workers. Finally, possibilities for further research and intervention aimed at promoting decent work through job crafting are discussed.

## Introduction: Decent Work and the Psychology of Working Theory Framework

The twenty-first century has been characterized by systemic shocks (September 11, 2001, attacks in the United States, the 2007–2008 global financial crisis) that have posed a wide array of challenges for the world of work (Blustein et al., 2019a; Johnson et al., 2020). The COVID-19 pandemic has exacerbated these challenges, accelerating trends that were already underway, such as insecurity, instability, and continuous changes to work contexts, with a dramatic decline in the number and quality of accessible jobs (Blustein et al., 2020; Kniffin et al., 2021). The people at greater risk are those already vulnerable because of their health, economic, or social conditions, including the old, the young, people in precarious employment, the unemployed, women, ethnic minorities, and people with disabilities (Tamin et al., 2021). In this scenario, effective strategies needed for long-lasting recovery from the current crisis should not solely aim for a “return to normal” but rather strive to change policies and work practices that damage and diminish vulnerable workers (Blustein et al., 2020).

Consistent with this principle, in the last two decades, Blustein and his colleagues have advanced the psychology of working theory (PWT; Blustein, 2013; Duffy et al., 2016; Blustein et al., 2019b), an elaboration of the psychology of working framework (Blustein, 2006), to align the contemporary working milieu with a social justice agenda (Blustein et al., 2019a). The leading objective of the PWT is to promote greater social inclusion (i.e., inclusivity) and access to decent work for everyone (Di Fabio and Blustein, 2016; Blustein et al., 2019a; Di Fabio and Kenny, 2019).

In this framework, we can also underline the contribution of Howard E. Gardner’s studies (Gardner et al., 2001) as he provided the definition of good work (i.e., a job that is engaging, excellent, and ethical) as well as the pioneering contribution of the two-factor model of work motivation developed by Herzberg (1966). Two arrays of needs affect job satisfaction in his perspective: motivation factors (i.e., achievement, recognition, the work itself, responsibility, advancement, and the possibility for growth) and hygiene factors (i.e., company policies and administration, relationship with supervisors, interpersonal relations, working conditions, and salary).

Nowadays, decent work in the PWT approach (Blustein et al., 2019a) is currently defined by five job characteristics: (1) physically and psychologically safe working conditions, (2) adequate compensation, (3) sufficient rest/free time, (4) organizational values that incorporate family and social values, and (5) reasonable access to healthcare (Duffy et al., 2016). In this approach, decent work is the primary determinant of work fulfillment (job satisfaction and work meaning) and wellbeing by achieving three arrays of basic human needs: (1) survival, which involves essential resources (e.g., food, shelter, and healthcare); (2) social connections/contributions, which reflect the need to connect and contribute to a larger community; and (3) self-determination, or the need for human behaviors to align with authentic, meaningful goals (Duffy et al., 2016). Starting from these premises, PWT researchers introduced a model (Duffy et al., 2018) to examine predictors and outcomes of decent work. In their model, decent work is the central variable, with contextual variables (i.e., economic constraints and marginalization) as principal predictors, and wellbeing both within and beyond work as outcomes (Duffy et al., 2018). The model encompasses four moderators of links between the above three variables: proactive personality, social support, critical consciousness, and economic conditions. Furthermore, two variables mediate the seeking of decent work, namely work volition (an individual’s perceived freedom to make occupational choices) and career adaptability (an individual’s capacity to use psychosocial support to project and achieve work-based goals; Duffy et al., 2018; Blustein et al., 2019a). The decent work agenda has called upon vocational psychologists to advance new ideas and initiatives for psychological research to develop decent work and inclusiveness for vulnerable workers (Blustein et al., 2016, 2020). Job crafting (e.g., Wrzesniewski and Dutton, 2001; Bakker et al., 2012) represents a promising way to achieve this. Job crafting refers to a bottom-up process that employees undertake themselves to better match their own needs, aspirations, preferences, and circumstances to their jobs (Wrzesniewski and Dutton, 2001).

Despite the growing interest in job crafting as an antecedent to organizational change, no study has addressed how job crafting can advance decent work. Thus, the purpose of this perspective article is to analyze the concept of job crafting in three new ways: (1) by identifying which components of the PWT model (basic needs, career adaptability, and proactive personality) can be associated with job crafting, (2) by examining job crafting interventions in vulnerable workers, and (3) by discussing whether job crafting could shape decent work for vulnerable workers.

## Job Crafting: Evolution of the Concept

Job crafting has generally been approached from two perspectives (Demerouti, 2014). The first perspective, developed by Wrzesniewski and Dutton (2001), defines job crafting as employee-driven cognitive and physical changes in work tasks and relational boundaries. Within this concept, there are three types of crafting: task crafting (shaping the number, aims, or kind of job tasks), relational crafting (modifying the social features and interactions of the job), and cognitive crafting (changing the way employees think about their jobs). The prime motivation behind job crafting is to achieve basic human needs for autonomy, positive self-image, and relatedness (Wrzesniewski and Dutton, 2001). To this end, employees switch from a “one-size-fits-all” job to a tailored job that is more aligned with their preferences, abilities, and needs (Wrzesniewski et al., 2013). By doing so, job crafting leads work activities to become a source of meaning and identity (Wrzesniewski and Dutton, 2001). Thus, workers can experience the related benefits of work meaningfulness, such as job satisfaction, thriving, and resilience (Berg et al., 2013).

Starting from the conceptualization proposed by Wrzesniewski and Dutton (2001), a variety of researchers have expanded this model (Rudolph et al., 2017). Among them, Leana et al. (2009) introduced collaborative crafting, which consists of the joint effort of workers to customize their jobs together.

The second perspective relates job crafting to the job demands–resources model (JD-R; Bakker and Demerouti, 2007). Job demands concern all aspects of the job that require effort from employees, while job resources deal with all the features of the job that are optimal for fulfilling work goals, decreasing job demands, and promoting personal development (Demerouti et al., 2001). In this vein, Tims and Bakker (2010) defined job crafting as changes undertaken by employees to balance their job demands and job resources with their abilities and needs. Achievement of fit, in turn, leads to greater job satisfaction, perceived meaningfulness of work, and work engagement (Tims and Bakker, 2010). Tims and her colleagues captured four empirical dimensions of job crafting (Tims et al., 2012): (1) increasing challenging job demands (i.e., crafting work tasks to learn and to achieve goals), (2) decreasing hindering job demands (i.e., shaping tasks that interfere with workers’ personal growth and goals), (3) increasing structural job resources (i.e., discovering new avenues for professional development and autonomy), and (4) increasing social job resources (i.e., asking for support from supervisors/colleagues or feedback/coaching; Tims et al., 2012).

Subsequently, Petrou et al. (2012) collapsed the two dimensions by Tims et al. (2012) related to job resources (increasing structural and social job resources) and differentiated between three characteristics of job crafting: seeking resources, seeking challenges, and reducing demands. In a similar way, Lichtenthaler and Fischbach (2016) aggregated the definition by Tims et al. (2012) into the “increasing” factors of job demands in promotion-focused job crafting and the “decreasing” factors of job demands in prevention-focused job crafting. Lastly, Bruning and Campion (2018) combined the Wrzesniewski and Dutton (2001) perspective with the Tims et al. (2012) conceptualization and advanced a comprehensive taxonomy of job crafting activities encompassing role crafting and resource crafting (Bruning and Campion, 2018).

## Results of Job Crafting Research and the PWT Model of Decent Work

The majority of job crafting research has been concerned with individual and organizational performance (e.g., Petrou et al., 2015; Bakker et al., 2020; Boehnlein and Baum, 2020), as well as changes in employees’ work engagement (e.g., Oprea et al., 2019), self-efficacy (e.g., Tims et al., 2014; Miraglia et al., 2017), and wellbeing (e.g., Peral and Geldenhuys, 2016; Wang et al., 2020). However, in considering results from job crafting research, we focused on those pieces of evidence relevant to the PWT model (Duffy et al., 2018; Blustein et al., 2019a). We examined psychological variables encompassed in the definition of decent work, namely the need for self-determination and meaningful work, as well as the psychological moderators of the PWT model and mediators of seeking decent work, specifically proactive personality, career adaptability, and work volition (Duffy et al., 2018; Blustein et al., 2019a).

Concerning self-determination, Bakker and Oerlemans (2019) found that daily job crafting is predictive of an employee’s momentary satisfaction of self-determination needs (i.e., autonomy, competence, and relatedness). More specifically, Hornung (2019) showed that task crafting predicted satisfaction of all self-determination needs (autonomy, meaning, competence, and impact), whereas cognitive crafting predicted satisfaction of only meaning and competence. Furthermore, a study of the literature found that employees craft their jobs to satisfy their self-determination needs, in particular, those related to autonomous intrinsic motivations (i.e., to make work more enjoyable and challenging; Lee and Song, 2019; Shin and Jung, 2019).

There is less literature on job crafting and meaningful work (Tims et al., 2016; Petrou et al., 2017). A first study reported that job crafting had an indirect effect on meaningful work, showing a positive association with demands–abilities fit that, in turn, leads to more meaningfulness (Tims et al., 2016). A second study observed that job crafting by increasing structural resources was positively associated with meaning-making only when occupational role salience was high (Petrou et al., 2017). Conversely, when job crafting opportunities were low, leisure crafting (i.e., alternative crafting that employees apply in their free time to compensate for needs not satisfied at work) was positively related to meaning-making (Petrou et al., 2017).

Meta-analytic results (Rudolph et al., 2017) indicated that proactive personality had a strong positive association with job crafting, confirming that employees characterized by proactive personalities were most inclined to craft their jobs (Bakker et al., 2012). Furthermore, job crafting mediated the positive association between proactive personality and mental health among workers (Zhang et al., 2018).

Another recent piece of the literature has investigated the association between job crafting and career adaptability (Federici et al., 2019; Woo, 2020). Correlational results indicated a positive relationship between career adaptability and job crafting (Woo, 2020). Moreover, Federici et al. (2019) found that job crafting moderated the relationship between career adaptability and work engagement in high-performance work practices.

No study, to our knowledge, has directly examined the relationship between job crafting and work volition, though the literature does contain results on job crafting and psychological capital, an antecedent of work volition (Cheung et al., 2020). According to these results, job crafting positively influenced the development of employees’ psychological capital (Kerksieck et al., 2019) and predicted psychological capital over time (Vogt et al., 2016).

## Results of Job Crafting Research in Vulnerable Workers

Over the last few years, job crafting research has provided findings on vulnerable workers, particularly older workers (Nagy et al., 2019; Zacher and Rudolph, 2019; Kooij et al., 2020), the unemployed (Hulshof et al., 2020a,b), workers with disabilities (Brucker and Sundar, 2020), and migrant workers (Arasli et al., 2019).

Regarding older workers, a pioneering article by Truxillo et al. (2012) highlighted the need to apply new life span development perspectives to the complex interaction between job characteristics and age. In this framework, they advanced a series of propositions to define job characteristics that facilitate aging at work (Truxillo et al., 2012). For example, they identified task significance, autonomy, and feedback from the job as aspects potentially related to engagement, satisfaction, and performance by older workers (Truxillo et al., 2012). Subsequently, researchers advanced that job crafting could help older workers fulfill these characteristics (Wong and Tetrick, 2017). They identified cognitive crafting as a promising strategy to increase the likelihood of person–job fit in older workers (Wong and Tetrick, 2017). Expanding these results, other scholars have examined the effects of different types of crafting in late-career employees (Lichtenthaler and Fischbach, 2016; Kooij et al., 2020). Lichtenthaler and Fischbach (2016) reported that only promotion-focused job crafting (i.e., increasing social/structural job resources and challenging job demands) keeps older employees motivated to continue working beyond retirement age. Kooij et al. (2020) investigated specific types of job crafting in older workers. They advanced “interest crafting” (autonomous changes to make a job more satisfying) and “work pressure crafting” (self-initiated changes to decrease work pressure) and observed that when workdays (days with greater work pressure and greater autonomy) were activated, daily interest crafting was positively related to daily work engagement and daily job performance (Kooij et al., 2020). On the other hand, work pressure crafting was negatively associated with daily work engagement and job performance (Kooij et al., 2020). Further results (e.g., Nagy et al., 2019; Zacher and Rudolph, 2019) have analyzed the relationship between job crafting and psychological processes related to aging at work. Nagy et al. (2019) observed that when late-career employees who perceived themselves as being subjectively younger engaged in job crafting, it enhanced the meaning of their work. Zacher and Rudolph (2019) built upon these results, clarifying that the association between subjective age and job crafting was mediated by a more open-ended occupational future time perspective.

Hulshof et al. (2020a,b) provided new insights into the role of job crafting in the unemployed. First, they introduced the new construct of reemployment crafting, adapting the conceptualization of Petrou et al. (2012) to the unemployed by adding seeking resources, seeking challenges, and reducing demands (Hulshof et al., 2020a). Subsequently, the authors investigated the effects of reemployment crafting on job search performance (Hulshof et al., 2020a). In that study, reemployment crafting was positively related to job search performance, whereas only challenge seeking was positively related to networking quality over time through a mediational effect by career exploration (Hulshof et al., 2020a). Lastly, a further study tested the efficacy of a job search demands–resources (JSD-R) intervention (i.e., reemployment crafting plus psychological capital interventions) in the unemployed (Hulshof et al., 2020b). Compared to the control group, unemployed people who received the JSD-R intervention showed higher levels of wellbeing, job search behaviors, motivation to undertake activities, subjective goal attainment, career exploration, and networking behaviors (Hulshof et al., 2020b).

With regard to workers with disabilities, Srivastava and Chamberlain (2005) highlighted those factors that organizations can use to incentivize workers with disabilities. Among them, these scholars suggested that shaping the jobs of disabled workers to align their tasks with their needs and abilities could be an effective strategy (Srivastava and Chamberlain, 2005). Similarly, Demerouti (2014) suggested job crafting as a functional strategy to retain and empower these workers. Nevertheless, only preliminary data are available on job crafting in workers with disabilities. Brucker and Sundar (2020) conducted a national survey highlighting that disabled workers are generally less prone to job crafting than those without disabilities (Brucker and Sundar, 2020). Specifically, workers with both disabilities and higher educational levels were more likely to engage in job crafting, whereas those with mobility limitations had the lowest probability of job crafting (Brucker and Sundar, 2020).

The last piece of evidence for vulnerable workers is given by a study exploring the influence of job crafting on migrant workers (Arasli et al., 2019). The findings from Arasli et al. (2019) showed that all job crafting dimensions had a positive effect on migrant employees’ job embeddedness. However, only task and cognitive crafting were found to have a positive effect on psychological capital (Arasli et al., 2019).

## Discussion: Can Job Crafting Shape Decent Work for Vulnerable Workers?

The decent work agenda (Blustein et al., 2019a,b) has called upon vocational psychologists to advance new research and intervention to promote work as a human right. As job crafting is an intervention that facilitates self-initiated changes made by employees to align their jobs with their needs, abilities, and preferences, it has the potential to shape decent work. While the literature has focused on job crafting by balancing job demands and resources consistently with economic principles, some results are noteworthy. First, job crafting leads employees to achieve greater self-determination and meaning at work (e.g., Bakker and Oerlemans, 2019; Hornung, 2019), two job characteristics of decent work. Second, the proactive personality is an individual characteristic that facilitates job crafting (e.g., Rudolph et al., 2017), as well as the achievement of wellbeing through decent work. Third, job crafting is mediated by career adaptability (e.g., Federici et al., 2019), as is seeking decent work. Fourth, job crafting indirectly enhances work volition (Vogt et al., 2016; Kerksieck et al., 2019; Cheung et al., 2020), the second mediator involved in seeking decent work. Thus, job crafting seems to help workers achieve job characteristics dealing with decent work, specifically those related to self-determination. Moreover, job crafting and decent work share common individual characteristics, making it possible to create common pathways of assessment and intervention. Examining the added contribution that our perspective could introduce for research and intervention in vulnerable workers, it is important to further consider job crafting and decent work not only as connected by common individual characteristics but also as embedded process of personal and social accomplishment. If specific variables emerged as critical, the lived work experience of each person has to be taken into account, since job demands and job resources refer to complex socio-material conditions, requiring a context-driven approach to better detect and analyze them. Additional considerations arise from recent studies. Organizational goals could generate opportunities for vulnerable workers because clear expectations and development inducements enhanced vulnerable workers’ employability competencies (Audenaert et al., 2020). From another point of view, recent research suggested that decent work plays a significant role in promoting a positive approach to work, showing psychological capital as a mediating variable in promoting autonomous work motivation (Ferraro et al., 2018).

In conclusion, regarding job crafting and decent work, our perspective suggests that job crafting could be considered in a new light, in terms of interventions not only to enhance organizational performance but also to shape decent work. Furthermore, the recent literature has shown promising results on job crafting in vulnerable workers. For example, job crafting in older employees predicted greater meaning at work, and job crafting interventions in the unemployed predicted more job searches and more motivation to undertake activities (Nagy et al., 2019; Hulshof et al., 2020b). Conversely, empirical evidence on job crafting in workers with disabilities and migrant employees is minimal (Arasli et al., 2019; Brucker and Sundar, 2020). However, these results indicate the potential to promote decent work in vulnerable workers by crafting their jobs. In this promising new perspective, job crafting might focus on common variables shared by job crafting and decent work, starting with self-determination, meaning at work, career adaptability, and work volition. Hence, we advance a new potential mission for the job crafting framework: decent work and inclusiveness as a human right (Blustein et al., 2019a,b).

## Data Availability Statement

The original contributions presented in the study are included in the article/supplementary material, and further inquiries can be directed to the corresponding author.

## Author Contributions

AS wrote the first draft of the paper. ADF conceptualized the paper, supervised and tutored AS, and reviewed, edited, and wrote the final draft of the paper. All the authors contributed to the article and approved the submitted version.

### Conflict of Interest

The authors declare that the research was conducted in the absence of any commercial or financial relationships that could be construed as a potential conflict of interest.
